# The relevance of oral food challenge in a patient allergic to peanut and tree nuts

**DOI:** 10.5415/apallergy.0000000000000109

**Published:** 2023-09-07

**Authors:** Rita Limão, Borja Bartolomé, Fátima Cabral Duarte

**Affiliations:** 1 Immunoallergology Department, Centro Hospitalar Universitário Lisboa Norte, Lisboa, Portugal; 2 Research and Development Department, Roxall España, Bilbao, Spain

**Keywords:** Allergy, oral food challenge, peanut, skin prick test, tree nuts

## Abstract

Peanut allergy is one of the most common food allergies in childhood. In vitro cross-sensitization between peanut and tree nuts (TN) is high, but only a subgroup of patients allergic to peanut will have a concomitant allergy to one or several TN. In this article, the authors report a case of a 12-year-old boy who experienced 1 episode of lips and mouth itching, generalized urticarial, and eyelid angioedema 20 minutes after ingestion of peanut at 4 years of age. The immunoallergological study revealed the presence of a concomitant allergy to peanut, pistachio, and cashew confirmed with medically supervised oral food challenges (OFC) in a child who had never eaten these TN. The mechanism of IgE-mediated hypersensitivity was demonstrated by positive skin prick tests (SPT) with commercial extracts, although the specific IgE (sIgE) for these foods was negative. As described in the literature, we concluded that serum peanut and TN sIgE measurements have lower sensitivity than SPT to assess IgE sensitization, and OFC is the gold standard for accurate diagnosis of food allergy. We highlight the relevance of excluding or confirming TN allergy in a peanut-allergic patient who had never ingested certain TN, and of knowing the clinical relevant cross-reactivity patterns between TN, pistachio/cashew, and walnut/pecan, that could reduce the need for OFC in clinical practice, reducing allergy rates and financial and health burdens of food allergy.

## Introduction

Peanut allergy is one of the most common food allergies in childhood and is often lifelong [1-3]. Unless there is a high pretest probability of peanut allergy (convincing history, background atopy, and a high level of peanut sensitization), peanut reactions should be documented by oral food challenges (OFC) as a positive skin test or serum-specific IgE (sIgE) is not always associated with clinical reactivity [3, 4].

In vitro cross-reactivity between peanut and tree nuts (TN) is high [5], but only a subgroup of patients will have a concomitant allergy to peanut and TN [6].

In this article, we present a case of IgE-mediated peanut allergy in which skin prick tests (SPT) and sIgE results were discrepant, and the presence of cross-sensitization and reactivity between cashew and pistachio, TN never eaten by the patient, was confirmed by in vitro and in vivo tests.

## Case report

A 12-year-old boy experienced an episode of lips and mouth itching, generalized urticarial, and eyelid angioedema 20 minutes after ingestion of peanut at 4 years of age. There was no digestive, pulmonary, or cardiovascular involvement. The symptoms resolved 30 minutes after taking oral hydroxyzine.

The patient had prior tolerance to peanut and, since the described episode, he tolerated the ingestion of other legumes such as soy, peas and grains, as well as some TN, specifically almonds, hazelnuts, pine nuts, and chestnuts. He has never tried to eat other TN.

Personal history includes mild atopic eczema since 3 years old, well controlled with emollients, mild allergic rhinitis, and asthma with mite sensitization since the age of 4 years, currently not requiring daily therapy. Food diversification started at 4 months of age, with no reported complications. Father has allergic rhinitis and no family history of food allergy was reported.

In an Allergy Food appointment, we performed SPT with commercial extracts (Roxall-Aristegui) and prick-prick test with fresh foods. Positive results (wheal ≥3 mm compared with the negative control) were obtained with peanut, pistachio, and cashew extract (Table 1) [7].

**Table 1.** Skin prick tests and sIgE to peanut and tree nuts

	Commercial extract*	Fresh food*	sIgE, kU_A_/L
Histamine, 10 mg/mL	7	NP	-
NaCl 0.9%	0	0	-
Peanut	6	NP	<0.10
Hazelnut	0	0	<0.10
Almond	0	0	<0.10
Pistachio	6	NP	<0.10
Cashew	7	NP	<0.10
Pine nut	NP	0	<0.10
Walnut	0	0	<0.10
Chestnut	0	0	<0.10

NP, not performed; sIgE, specific IgE.

* Mean wheal diameter (mm).

Serum-sIgE levels to peanut and TN extracts using ImmunoCAP method (ThermoFisher Scientific) were <0.10 kU_A_/L (Table 1), as well as against molecular components *rAra h 1*, *rAra h 2*, *rAra h 8*, *rAra h 9*.

A medically supervised open-labeled OFC with roasted peanut was performed and 15 minutes after the cumulative dose of 3.33 g (10 whole peanut kernels), skin itching, limb urticarial, and facial angioedema were observed (Table 2) [8].

**Table 2.** Oral food challenge protocols

		Peanut kernel (whole)	Pistachio (whole)	Cashew (whole)	Walnut (halves)
1st	Doses are given 15 min apart	¼	½	¼	¼
2nd		1	2	½	½
3rd		2	3	1	1
4th		3	4	2	2
5th		4	5	3	3
6th		5	6	4	4
Cumulative dose		15 peanut kernel ~5 g	20 whole nuts ~5 g	10 whole nuts ~5 g	10 halves ~5 g

In order to introduce the TN that has not been eaten by the child, open-labeled OFC with pistachio, cashew and walnut were performed. He developed mouth itching, generalized urticarial, and eyelid angioedema 20 minutes after the intake of 20 units of pistachio and 10 units of cashew (cumulative doses of ≈5 g). The walnut OFC was negative (Table 2) [8].

**Figure 1.**
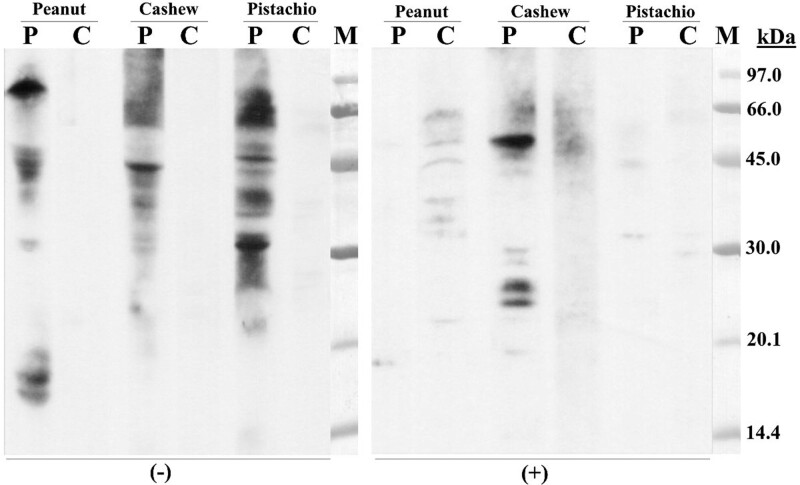
SDS-PAGE immunoblotting. Lane P: patient serum; Lane C: control serum (pool of sera from nonatopic subjects); Lane M: molecular mass standard. (−), without 2-mercaptoethanol; (+), with 2-mercaptoethanol.

An SDS-PAGE immunoblotting assay according to Laemmli [9] under nonreducing (without 2-mercaptoethanol) and reducing conditions (with 2-mercaptoethanol) was carried out with peanut, pistachio, and cashew extracts. Some IgE-reactive bands were detected in the 3 extracts in nonreducing conditions, whereas only in cashew extract were observed bands in reducing ones: bands of ≈50, 26, 25 kDa (Fig. 1). The 50 kDa band could be *Ana o 1* and the bands of 26/25 kDa could be the protein *Ana o 2*. In nonreducing conditions, an IgE-reactive band of ≈17 kDa detected in peanut extract could be *Ara h 2*.

The patient maintains avoidance of peanut, pistachio, and cashew, and an anaphylaxis action plan was provided. No accidental allergic reactions have been observed.

We have obtained the consent from the patient’s legal representative for the publication of his case report. Ethical committee approval was not necessary in this case.

## Discussion

We present a child who developed an immediate reaction 15 to 20 minutes after peanut ingestion, where sensitization may have occurred through an impaired skin barrier (atopic dermatitis) or orally (previous ingestion of peanut) [10].

In our patient, peanut sensitization was confirmed by a positive SPT with commercial extract despite having negative sIgE for peanut and its molecular components. This is not surprising as skin tests have shown greater sensitivity relative to sIgE assays in diagnosing peanut allergy in pediatric studies [2]. Similarly, while *Ara h 2* had the greatest specificity in confirming the diagnosis, it had lower sensitivity when compared with SPT or sIgE [3, 11, 12]. In this case, a positive OFC confirmed peanut allergy diagnosis.

Cross-sensitization may exist between peanut and TN and among TN, although such patterns do not always result in clinically relevant allergy [13]. Another concern associated with the decision to introduce TN in patients allergic to peanut is the potential for cross-contamination, and some patients are not able to distinguish between different TN [14]. Then in peanut-allergic patients who have never ingested TN, there may be hesitancy to introduce TN at home.

Our patient had never tried pistachio, cashew, and walnut. The SPT were positive to pistachio and cashew and negative to walnut. Indeed, allergy clusters have been described: cashew/pistachio and walnut/pecan, where cashew and walnut seem to be the dominant allergens. As sensitization assessment by SPT and/or sIgE before TN introduction remains susceptible to false-positive results and could delay TN diet introduction [5, 14], recently it has been proposed that patients suspected of high probability of cashew/pistachio allergy based on the clinical testing should be first challenged with pistachio, noting that if a positive result were obtained, there is a high chance he would also be cashew allergic, what was confirmed in our patient by OFC. In the same way, in patients with low probability for walnut/pecan allergy based on the clinical testing, challenge should be first to walnut, and if tolerant, the patient likelihood of pecan allergy is near zero, what can be inferred in our patient after a negative walnut OFC [15]. Using these diagnostic algorithms, a reduction in need for OFC can be enabled, which, despite being diagnostic gold standard of food allergy, are time and resource intensive, limited in availability in many tertiary allergy services, and exposes the child to the risk of severe reactions [15, 16].

The SDS-PAGE immunoblotting using the patient´s serum detected sIgE-reactive proteins in peanut, cashew, and pistachio extracts, which can justify an allergic reaction through the ingestion of these foods (Fig. 1).

sIgE reaction to proteins from peanut and pistachio was clearly affected by the 2-mercaptoethanol treatment, which breaks the disulfide bonds that can be found in certain proteins responsible for maintaining their 3-dimensional structures. Likely the IgE reaction to these extracts depends on the 3-dimensional structure of the proteins.

The 17 kDa IgE-reactive band detected in peanut extract could be *Ara h 2*, the peanut allergen most associated with allergic symptoms after peanut ingestion [3].

In cashew extract, IgE-reactive bands with molecular weights of ≈50, 25, and 26 kDa were detected, and these bands could be *Ana o 1*, a 7S vicilin, and the small subunits of the protein *Ana o 2*, a 11S globulin [17].

*Ana o 1* and *Ana o 2* are 2 of the 3 major cashew allergens described, and *Ana o 2* is also referred to as anacardein [18]. Furthermore, cross-sensitization and reactivity has been described between *Pis v 3* from pistachio and *Ana o 1*, which is in agreement with the concomitant allergy to these 2 nuts that present our patient [13].

The remaining bands observed in immunoblotting and their molecular weights do not correspond to proteins identified in the allergen databases. In fact, molecular mass of the bands detected in the electrophoresis without 2-mercaptoethanol is not easily related to the database molecular masses of the described allergens, which represents a limitation of the study. The relationship is more direct with the molecular masses obtained in electrophoresis under standard conditions of the technique (with 2-mercaptoethanol).

Current practice in many centers is avoidance of all nuts in children with a known nut allergy, which negatively impacts the quality of life of children and their families and carries a potential risk of developing nut allergy due to the lack of exposure [17, 19].

This case report highlights the possible discrepancy between diagnostic methods: negative sIgE levels but positive results with SPT and detection of IgE-binding proteins in immunoblotting with peanut, pistachio, and cashew extracts, and food allergy confirmed by OFC. Furthermore, it confirmed clinically relevant cross-reactivity between pistachio and cashew, which may reduce the need for OFC in clinical practice. Finally, the exclusion of allergy to certain TN in a patient with peanut, pistachio, and cashew allergy reduces allergy rates and financial and health burdens of food allergy, which is an important consideration given increasing allergy prevalence worldwide.

## Conflicts of Interest

The authors have no financial conflicts of interest.
